# Signal Intensities Derived from Different NMR Probes and Parameters Contribute to Variations in Quantification of Metabolites

**DOI:** 10.1371/journal.pone.0085732

**Published:** 2014-01-21

**Authors:** Paige Lacy, Ryan T. McKay, Michael Finkel, Alla Karnovsky, Scott Woehler, Michael J. Lewis, David Chang, Kathleen A. Stringer

**Affiliations:** 1 Pulmonary Research Group, Department of Medicine, University of Alberta, Edmonton, Alberta, Canada; 2 Department of Chemistry, University of Alberta, Edmonton, Alberta, Canada; 3 Department of Clinical, Social and Administrative Sciences, College of Pharmacy, University of Michigan, Ann Arbor, Michigan, United States of America; 4 Department of Computational Medicine and Bioinformatics, School of Medicine, University of Michigan, Ann Arbor, Michigan, United States of America; 5 Department of Medicinal Chemistry and the Biochemical Nuclear Magnetic Resonance Core, College of Pharmacy, University of Michigan, Ann Arbor, Michigan, United States of America; 6 Chenomx, Inc., Edmonton, Alberta, Canada; National Research Council of Italy, Italy

## Abstract

We discovered that serious issues could arise that may complicate interpretation of metabolomic data when identical samples are analyzed at more than one NMR facility, or using slightly different NMR parameters on the same instrument. This is important because cross-center validation metabolomics studies are essential for the reliable application of metabolomics to clinical biomarker discovery. To test the reproducibility of quantified metabolite data at multiple sites, technical replicates of urine samples were assayed by 1D-^1^H-NMR at the University of Alberta and the University of Michigan. Urine samples were obtained from healthy controls under a standard operating procedure for collection and processing. Subsequent analysis using standard statistical techniques revealed that quantitative data across sites can be achieved, but also that previously unrecognized NMR parameter differences can dramatically and widely perturb results. We present here a confirmed validation of NMR analysis at two sites, and report the range and magnitude that common NMR parameters involved in solvent suppression can have on quantitated metabolomics data. Specifically, saturation power levels greatly influenced peak height intensities in a frequency-dependent manner for a number of metabolites, which markedly impacted the quantification of metabolites. We also investigated other NMR parameters to determine their effects on further quantitative accuracy and precision. Collectively, these findings highlight the importance of and need for consistent use of NMR parameter settings within and across centers in order to generate reliable, reproducible quantified NMR metabolomics data.

## Introduction

^1^H Nuclear magnetic resonance (NMR) is widely used for metabolic studies of human biological samples [1], [2], [3], [4]. There have also been studies of the application of multi-dimensional [5] and multi-nuclear [6] NMR approaches in the field of metabolomics. NMR has an exceptional capacity in metabolomics because numerous metabolites can be rapidly identified and quantified from spectra acquired from body fluids such as serum, plasma, and urine and it has demonstrated utility in more difficult-to-obtain samples such as intact or tissue extracts, cerebral spinal fluid, and vitreous humour [7], [8]. NMR is a non-destructive, single assay technique that does not require sample derivatization or separation columns, and is particularly useful in recognizing as well as quantifying compounds, such as sugars, amino acids, and other relatively unreactive compounds, that are not easily detected or measured by other metabolomics approaches. Because NMR has the ability to identify and quantify hundreds, and potentially thousands, of small molecules, it greatly enhances our ability to rapidly characterize patterns of metabolites that may be associated with disease. Thus, quantitative analysis of NMR spectra of urine and blood samples, in combination with multivariate mathematical modeling, has been proposed as a novel approach for biomarker discovery for a number of illnesses including heart disease [9], pneumonia [10], cancer [11], [12], and acute lung injury [13], [14] to name a few recent examples. The majority of human NMR metabolomics studies to date have been carried out at different centers, by separate groups, and often all of the data involved in any single study have been collected on an individual instrument at a single location. Cross-site analytical validity studies have been conducted [15], [16], [17], but these used chemometric techniques rather than targeted profiling, which involves metabolite identification and quantification. The latter is essential for the clinical application of metabolomics-derived biomarker discovery, and is important since analytical parameters used for NMR spectral acquisition and analysis at different sites may vary, potentially producing different results in both the identification and quantification of metabolites.

Given the need and importance of sound validation practices for biomarker discovery [18], [19], [20] and limited cross-center analytic validation studies in NMR metabolomics, we determined the reproducibility of compound identification and quantification of urine metabolites from NMR spectra generated at two sites using technical replicate samples. Samples were obtained from healthy human donors at a single center, split into two aliquots, and data from the technical replicates were acquired at two separate NMR facilities. Initially, most metabolites measured in the replicate samples at the two sites strongly correlated. However, we encountered a surprising, unexpected peak suppression phenomenon that has not previously been reported for metabolite measurement in urine samples. Subsequent NMR data acquisition and rigorous testing of experimental parameters, optimization techniques, and hardware calibrations revealed an unexpected sensitivity dependence on the type of setup from which the final quantitative result was obtained. Here, we report our findings, give practical examples to assist in identification of potential problems, and make recommendations so that future studies avoid these relatively easily made errors in data collection and analysis.

## Methods and Materials

### Subjects, ethics statement and sample collection

Normal, healthy volunteers (20 donors, ≥50 years of age) were identified and recruited for the University of Michigan's Institutional Review Board (UM IRB) approved study (protocol number, HUM00038122) via the Claude D. Pepper Older Americans Independence Center (OAIC) Research Participant Program at the University of Michigan's Geriatric Center and the Michigan Institute of Clinical and Health Research (MICHR) clinical studies website (UMClinicalStudies.org). This was done in accordance with the ethical standards of the UM IRB and the Helsinki Declaration of 1975, and as revised in 2000 [21]. For study eligibility, subjects had to be non-smoking, and non-obese with no known medical conditions that required chronic drug therapy. On the day of sample collection, volunteers presented to MICHR's clinical research unit (http://www.michr.umich.edu). Following the acquisition of UM IRB-approved written informed consent, fasting (12 h) subjects provided a midstream clean-catch urine sample between 0830-0930 [22] which was collected and processed using a standard operating procedure (SOP) that was prospectively constructed and mutually agreed upon by the two analytical centers (see Text S1). Briefly, urine collection cups (Becton Dickinson (BD) Vacutainer, Franklin Lakes, NJ, USA) were coated with 100 µl NaN_3_ (10%) and allowed to dry before use. Samples were processed within 3 h of collection and following centrifugation (4°C), the addition of an internal standard (IS) solution (IS-1, Chenomx IS: DSS with added imidazole), and pH adjustment to 7.0±0.25, a portion of the sample (1 ml) was transferred to a microcentrifuge tube and dipsticked with a Chemstrip 10 MD (Roche Diagnostics, Indianapolis, IN, USA). Samples were then aliquotted (1 ml) into labeled sterile microcentrifuge tubes and frozen (−80°C) until the time of assay. Near to the time of assay, a frozen replicate sample from each patient was shipped on dry ice from the University of Michigan (Ann Arbor, MI USA) to the University of Alberta (Edmonton, Alberta, Canada) using a next-day express courier service that assured samples remained frozen during shipping (http://www.worldcourier.com/).

### NMR spectral acquisition and analysis

At the time of the receipt of samples in Edmonton, they were removed from dry ice and stored (−80°C) until the day of measurement. On the day of NMR data acquisition, samples at both sites were thawed at room temperature and pH was measured prior to transferring sample (750 µl) into a clean glass NMR tube (328-PP-7 or 528-PP-7; Wilmad Labglass, Vineland, NJ, USA for the Alberta and Michigan samples, respectively) for spectral collection. The NMR analyses at the University of Alberta (National High Field Nuclear Magnetic Resonance Centre, NANUC) and the University of Michigan occurred within one week of each other. One sample was inadvertently not included in the shipment to Alberta, which resulted in a total of 19 samples analyzed at each site.

1-D-^1^H-NMR spectra were acquired at the University of Michigan's Biochemical NMR Core Laboratory using the same pulse sequence as that used by NANUC on a Varian (now Agilent Inc. CA, USA) 11.74 Tesla (500 MHz) NMR spectrometer with VNMRS console operated by host software VNMRJ 3.2, and equipped with a 3 mm HX probe with Z-axis gradients. Automatic sample handling was performed by a Varian 7510-AS robotics system and controlled by the spectrometer host software. Data collected at the NANUC facility in Edmonton was done on an Oxford 14.09 Tesla (600 MHz) NMR spectrometer also with a VNMRS console but utilizing a 5 mm HX probe with Z-axis gradient coils. Host software included VNMRJ 2.2c (Linux RHEL 4u3) with heavy in-house modifications to operate the Varian 768-AS (automatic sample handling system).

NMR spectra were recorded using the first increment of a ^1^H,^1^H-NOESY (commonly referred to as a 1D-NOESY or METNOESY) [23], [24], [25]. The indirectly detected dimension was not acquired, *i.e.*, associated incremental delays were always zero. The standard NMR pulse sequence consisted of a 10 ms recovery delay, 990 ms saturation pulse of ∼80 Hz (γB_1_) induced field strength empirically centered on the water resonance, two calibrated 90° pulses (see NOESY references above for complete descriptions of the phase cycles), a mixing time of 100 ms, a final 90° pulse for transverse signal detection, and lastly an acquisition period of 4 seconds. The total time of the experiment (5 seconds) is critical in order to eliminate T_1_ relaxation effects on the Chenomx Software quantitative analysis [26]. Optimal excitation pulse widths were obtained either utilizing nutation theory [27] built into the BioPack VNMRJ 3.2 software package, or an array of pulse lengths (while operating at maximum safe power) to determine the 360° pulse null for water, and dividing by four to obtain the 90° optimum [28], [29]. This avoids off resonance and radiation damping issues [30], [31], [32]. Data were zero-filled to twice the original data set size and a 0.5 Hz line-broadening apodization was applied prior to analysis. The pulse sequence did not utilize the more recent default 45° phase shifted excitation pulse [33] commonly used in modern versions of the 1D-NOESY (Agilent BioPack/userlib tnnoesy.c) sequence or the pulsed field gradients during the recovery delay or mixing period due to distortions in sharp resonances such as the internal DSS line shape and chemical shift reference [23]. Spectra at both sites were acquired at a temperature of 295.45±0.3 K.

Following NMR data acquisition, spectra were site de-identified and submitted to Chenomx (http://www.chenomx.com/; Edmonton, Alberta, Canada) for peak identification and quantification. The initial comparisons of the quantitated data from each site revealed notable differences in creatinine and urea concentrations, but not other metabolites (Fig. S1). The 3 mm data originally acquired at the University of Michigan utilized a 120° excitation pulse (default setting in robotics sample handling software). Therefore, we acquired NMR data at the University of Michigan using a 5 mm probe to carefully explore ranges of common NMR pulse sequence parameters such as pulse widths, saturation pulse strength, saturation frequency, and gain settings. For this re-acquisition of data, a technical replicate of each of the 19 samples was thawed and transferred to a 5 mm NMR tube (528-PP-7, Wilmad). A spectrum of each sample was re-acquired at the University of Michigan as described above with the exception of the use of a 5 mm probe Agilent “One-probe”.

### NMR Pulse Sequence

The 1D-NOESY NMR pulse sequence consisted of a 10 ms recovery delay, 990 ms saturation pulse of ∼98 Hz induced field strength (γB_1_) empirically centered on the water resonance, two calibrated 96° pulses, a mixing time of 100 ms, a last 96° pulse for transverse signal detection, and finally an acquisition time of 4 seconds. All spectra were acquired at a temperature of 295.45±0.3 K and were submitted to Chenomx, Inc. (Edmonton, Alberta) for peak identification and quantification. The slightly longer excitation pulse width was a standard setting used in the automation software and is not expected to perturb quantitation results.

### Verification of NMR probe size-dependent regional peak suppression

#### Sample preparation

A replicate of each of two urine samples was used for a series of verification experiments based on quantified urea concentration; one with the lowest and the other with the highest quantified urea concentration. Furthermore, two additional single-metabolite test solutions were generated by dissolving creatinine (Acros Organics, New Jersey, USA) in deuterium oxide (D_2_O; >99.8%, Acros Organics, NJ, USA) which was then diluted to a final concentration of 2 mM in either 100% D_2_O or 10% D_2_O with 10% Chenomx IS, DSS (0.5 mM final concentration).

#### 1H-NMR spectral acquisition and analysis

NMR spectra of these samples were acquired at the University of Michigan as described above using both the 3 mm and 5 mm probes with adjustments to the 1D-NOESY pulse sequence, as follows: The water signal was suppressed by pre-saturating the sample with an induced field (γB_1_) of 20 Hz, 78 Hz or 195 Hz for 990 ms and during the mixing time of 100 ms. The receiver gain was set to remain constant at 6 dB (to prevent receiver overload and/or analog to digital converter errors), with the exception of one experiment on each probe for which the receiver gain was set to 18 dB. Spectra for this verification were acquired at a temperature of 295.45±0.3 K.

### Spectral processing and metabolite identification and quantification

The spectra acquired from the 19 urine samples on the 3 and 5 mm probes at the University of Michigan and the 5 mm probe at the University of Alberta were processed and *.cnx files were generated by Chenomx using the Processor module in Chenomx NMR Suite 7.5 [34] which permits phase, baseline and shim correction. Compounds were identified and quantified using the Profiler module in the software which accounts for pH and references to the IS, DSS, and utilizes the Chenomx Compound Library containing 304 compounds. Metabolite concentrations were corrected for dilution secondary to the addition of the IS.

The spectra generated by the verification samples were all manually assessed using VNMRJ 3.2A. Peak heights relative to a drift corrected baseline were measured assuming a Lorentzian line-shape. This assumption is only valid when peaks are symmetric and well shimmed. Spectra were zero-filled to twice the original data set size and a 0.25 Hz line broadening weighting function applied to match the digital resolution. Each individual spectrum was manually checked for IS symmetry (methyl group of DSS at 0 PPM) and line width at 50%, 0.55%, and 0.11% total height, respectively. All spectra were found to be symmetric and conformed to our requirements of line width <1 Hz, <12 Hz, and <20 Hz for the three respective peak heights described above. Peak heights were observed relative to DSS and the effect of various parameter changes assessed. Parameter changes that were tested included: pulse carrier frequency, saturation frequency, saturation power, gain [30], and excitation pulse widths. These parameters were tested on both the 3 mm and 5 mm probes at the University of Michigan's Biochemical NMR Core. Identical parameters were tested on spectrometers equipped with 5 mm probes at the University of Alberta. Additionally, a single representative urine sample was transported on dry ice to the University of Toronto for assay on a 500 MHz Agilent spectrometer equipped with 3 mm ‘One-Probe’ in order to confirm the 3 mm observations.

### Statistical Analysis

The resulting Chenomx software quantified NMR data sets were normalized by auto-scaling: the mean value of each metabolite concentration was subtracted from each individual concentration value of the respective metabolite and divided by the standard deviation of the mean for the metabolite [30], [35]. The overall minimum value was then subtracted from each data point so that all values were positive. The resulting normalized data (5 mm probe) from each site were compared by linear regression (Pearson's correlation) using Prism 5 (GraphPad Software, Inc., La Jolla, CA). In all cases, a two-tailed p value≤0.05 was considered significant [36], [37]. Correlation graphs were constructed using Prism and a radar plot was constructed in Microsoft Excel using the normalized data set. Box and whisker plots of metabolite concentration data from both sites were constructed using Prism.

## Results

**Figure 1:**
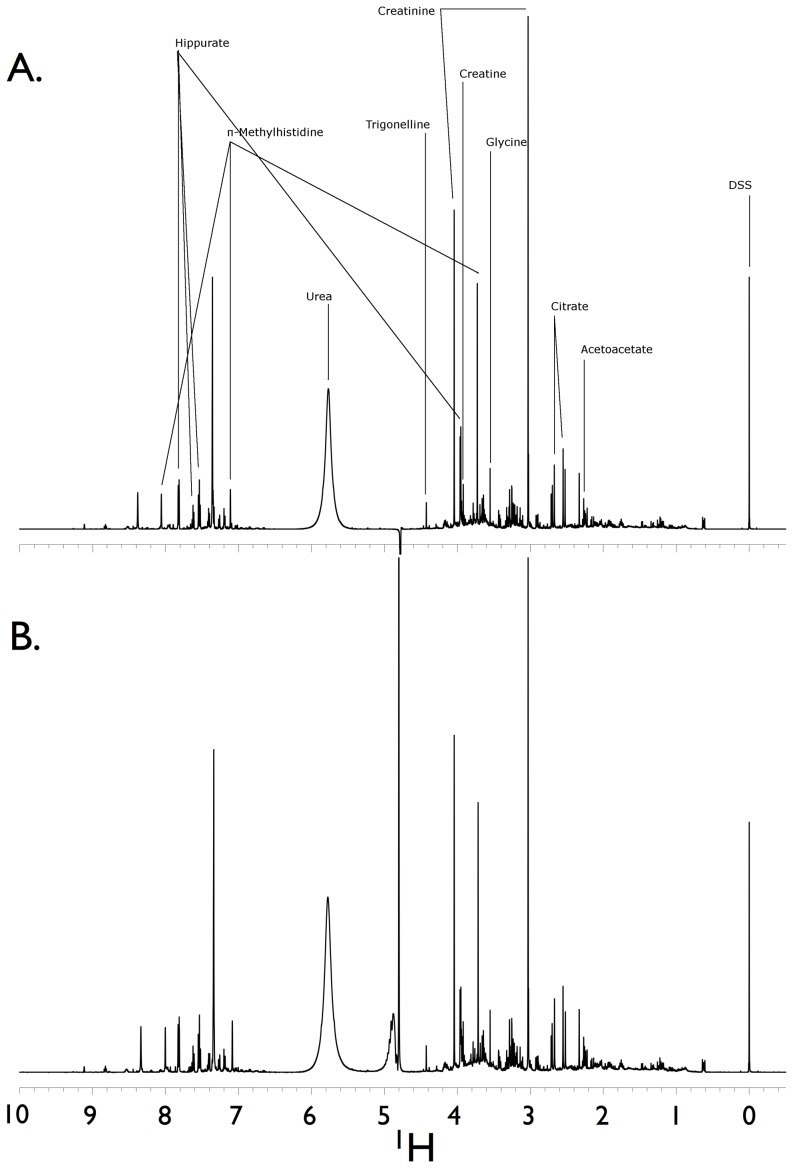
Hydrogen NMR spectra of two technical replicates of a single volunteer human urine sample. Data were collected on two separate NMR spectrometers. The first replicate (**A**) was run on a Varian VNMRS 600 MHz spectrometer equipped with 5 mm HX probe and a 768AS (automatic sample handling) robotic system at the University of Alberta. The second replicate (**B**) was run on a Varian VNMRS 500 MHz equipped with a 5 mm “One-probe” with Z-axis pulsed field gradients and an Agilent/Varian 7510-AS sample handling system at the University of Michigan's Biochemical NMR Core Laboratory. The induced saturation power or γB_1_ (and calibrated excitation pulses) for the 600 and 500 MHz spectrometer data was 80 Hz (90° pulse) and 98 Hz (96° pulse was the default robotic setting), respectively. A representative number of named metabolites and their assigned spectral peaks are shown as well as the internal standard, DSS. DSS = 4,4-dimethyl-4-silapentane-1-sulfonic acid.

**Figure 2:**
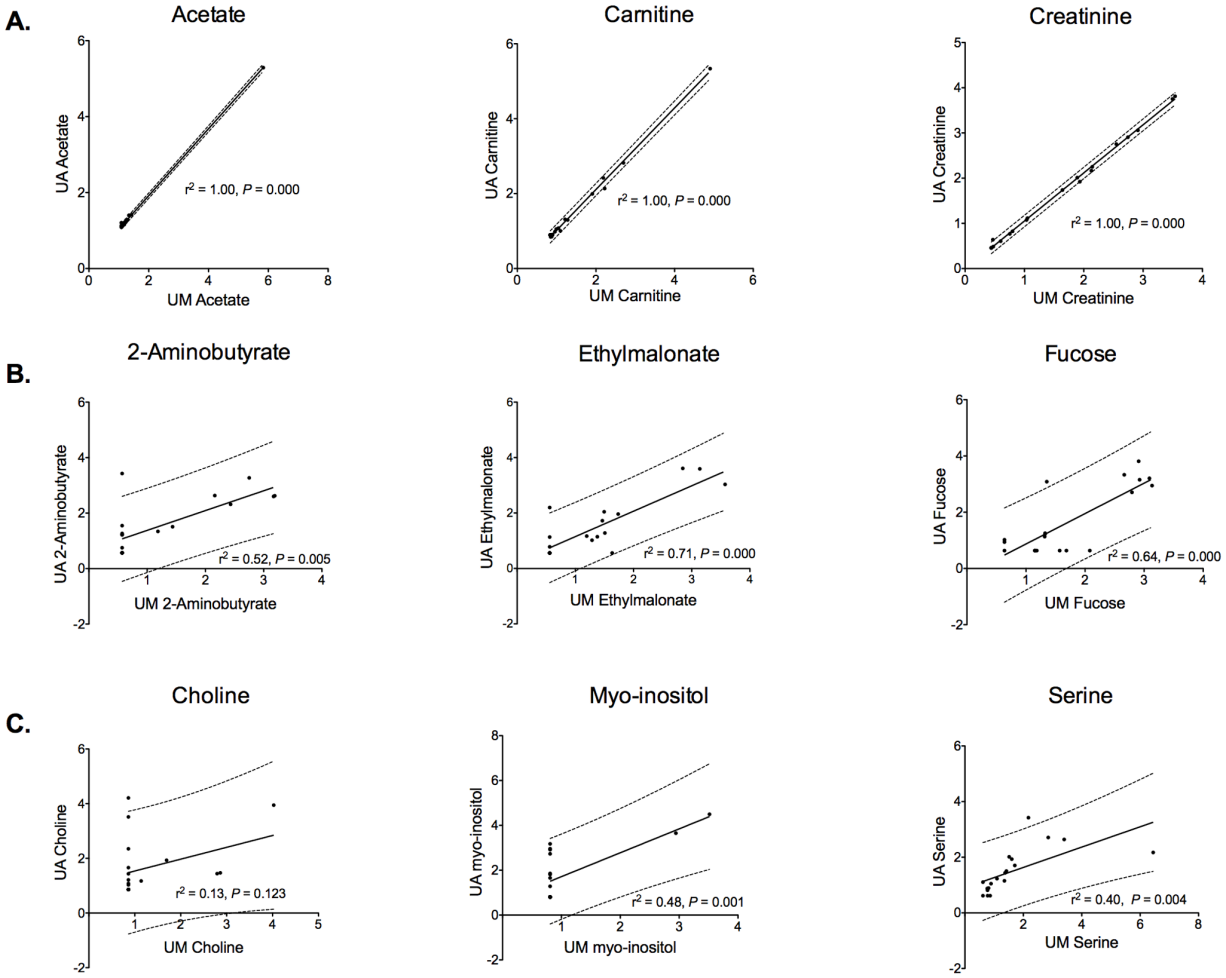
Representative Pearson linear regression plots with associated 95% prediction bands (dashed lines) of selected urine metabolites (normalized data). These data show: (**A**) a high degree of correlation (r^2^ = 1.0), acetate, carnitine, and creatinine; (**B**) a moderate degree of correlation (r^2^≥0.5, <0.9), 2-aminobutyrate, ethylmalonate, and fucose; and (**C**) a low degree of correlation (r^2^<0.5), choline, myo-inositol, and serine. All data shown are from ^1^H-NMR spectra acquired from technical replicate samples using 5 mm probes at the University of Alberta (UA) and the University of Michigan (UM). In all cases, the correlation p value was significant with the exception of choline (p = 0.123).

**Figure 3:**
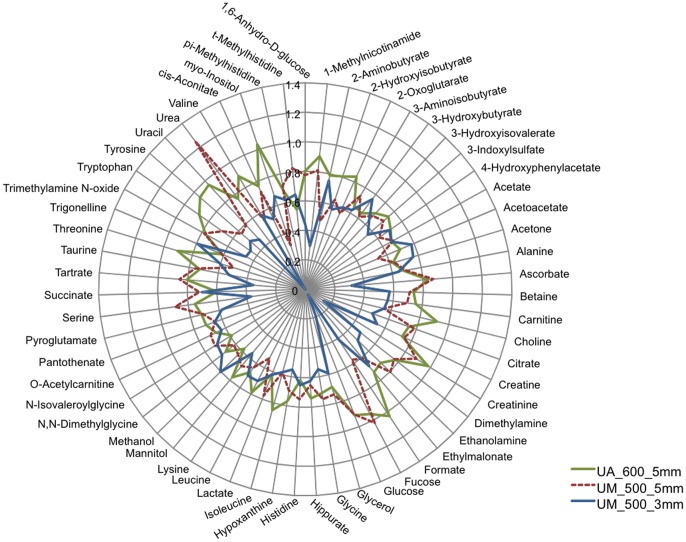
Radar plot of the normalized quantified ^1^H-NMR urine metabolites. The plot permits the visualization of the similarities and discrepancies between the data generated using a 5(University of Alberta (UA), and University of Michigan (UM)), and a 3 mm probe (UM). The data are the mean of the normalized values for each metabolite. Overall, the results from the 5 mm probes are more similar to each other than those from the 3 mm probe (also see Fig. S2 and Fig. S3).

**Figure 4:**
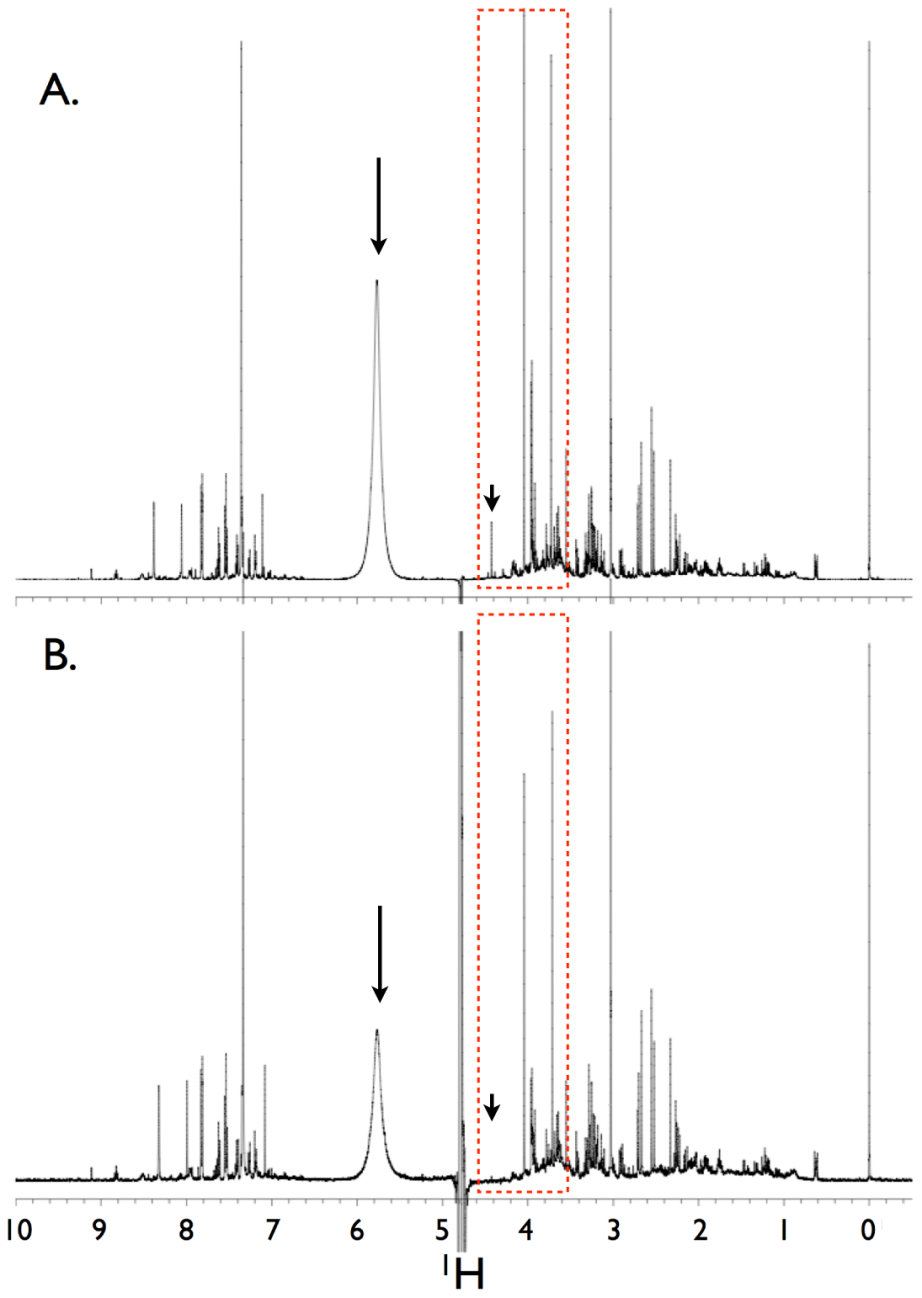
Effects of solvent suppression using two different probes on the same sample. The spectra were acquired utilizing the identical NMR pulse sequence as in Figure 1, with the exception that (**A**) was acquired using a 5 mm probe on a 600 MHz spectrometer at the University of Alberta and (**B**) was acquired using a 3 mm HX probe with Z-axis pulsed field gradients on a 500 MHz spectrometer at the University of Michigan. The decibel value of both settings for excitation and saturation were maintained between probes. However, due to probe design differences, the resulting pulse width for the 3 mm probe was 120° (same instrument decibel setting), and the saturation power for this probe resulted in a γB1-induced field of 226 Hz or 8 dB. The difference in metabolite intensities (*e.g.*, urea at ∼5.8 PPM, and sharp peaks at ∼4.0 and ∼3.75 PPM) in the same urine sample is readily evident. This inspired our extensive investigation to identify which experimental parameters were responsible for these differences, and to determine how the sensitivity of the metabolite resultants changed in response to several common NMR parameters (see text for details). The large arrows designate urea peaks, the small arrows indicate trigonelline (which was not observed in the 3 mm probe spectrum), and the red dashed-line boxes represent a region of the spectra that exhibited marked peak suppression, particularly around 4.5 PPM, near the water region.

**Figure 5:**
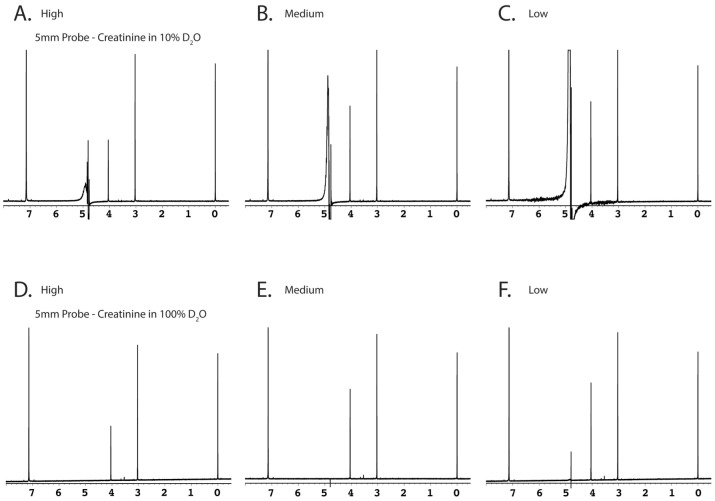
Comparison of changes in 1D-^1^H NMR spectra using different saturation powers. Samples were run utilizing either 5(**A–L**) or 3 mm probes (**M–X**). (**A–F**) and (**M–R**) show the effects of different saturation powers on synthetic creatinine (2 mM) dissolved in either 10% D_2_O (**A–C** and **M–O**), or >99.8% D_2_O (**D–F** and **P–R**). Two human volunteer urine samples were selected to represent a relatively high (**G–I** and **S–U**) and low urea concentration (**J–L** and **V–X**). Saturation powers yielding induced field strengths of 195 Hz, 80 Hz, and 20 Hz are shown in left, middle and right panels, respectively. Note the large increase in urea signal intensity (*e.g.*, **A** to **B**) when saturation power is just slightly diminished, indicating the amount of saturation transfer between exchanging atoms even in high D_2_O solvent conditions. The two samples with different urea concentrations were selected to elucidate the effect of urea/solvent exchange in a saturating system.

Urine samples were acquired and assayed from 19 healthy subjects (Table 1). A total of 59 metabolites were identified and quantified. Chemstrip and pH data for each sample are shown in Table S1. Spectra generated using comparable NMR spectrometers and 5 mm probes were similar despite subtle parameter setting differences at the two sites (Fig. 1). Quantification of the NMR spectra yielded metabolite concentrations that, as determined by linear regression of the normalized data, were similar (Fig. 2 and Fig. S2) with the exception of choline, a low abundance urine metabolite. Conversely, the quantified metabolite data from spectra acquired using a 3 mm probe (University of Michigan) using identical parameter settings were not as reliably consistent compared with those acquired using a 5 mm probe (Fig. 3, Fig. S1 and Fig. S3). This was most evident by differences in the concentrations of compounds determined by measuring resonances within ∼1 to 2 PPM of the solvent (water) carrier position (*i.e.*, 4.76, 4 and 3 PPM) such as creatinine (Fig. 4, Fig. S4 and Fig. S5). In an attempt to determine the source of this discrepancy we systematically re-acquired NMR signals using different parameter settings on the two probes. For example, we tested verification samples using a range of solvent saturation powers, carrier offsets, lock settings, and signal gain levels. The results showed an unexpected and dramatic suppression of peak intensity in a frequency-dependent manner. Specifically, metabolite NMR peaks closest to the solvent (water) suppression carrier position experienced more amplitude suppression than peaks farther away. We selected creatinine as an example because this molecule is often used to standardize urine metabolomics data [38], [39], [40], is usually present in relatively high concentrations in human urine, and has two sharp NMR singlets at 4.05 PPM and 3.03 PPM [3], [42]. Peaks below ∼3 PPM or above 7 PPM (Fig. 4 and Fig. 5A–X) demonstrated little significant intensity differences relative to the methyl group of the IS, DSS.

**Table 1:** Demographic characteristics of healthy control subjects.

Sample size (n)	19
Sex (%)	
Female	47
Age (mean ± S.D.)	54.4±8.1
Caucasian (%)	89.5%
Weight (kg; mean ± S.D.)	75.6±10.8
BMI^1^ (mean ± S.D.)	25.2±2.42

1 Body mass index.

We discovered that small, or even moderate values (deemed reasonable for common errors) in NMR parameters such as receiver gain, carrier frequency position, excitation pulse length, and spectrometer lock settings, had no statistically significant effects on spectral peak intensities relative to DSS. For example, setting the 90° excitation pulse length to 96° perturbed all resonances equally so that quantitative results comparing metabolites to the IS were indistinguishable to optimized data, albeit at overall reduced signal-to-noise ratio for a single scan. In addition, small purposeful changes (*e.g.*, 0.2 to 2 Hz) to simulate common setup errors in the optimal water suppression carrier position also resulted in no detectable metabolite quantitation errors. However, these small frequency changes from the optimal solvent suppression position resulted in substantial increases for the residual water peak intensity, as would be expected from the extensive solvent suppression literature.

To elucidate and simplify our initial observations originating from complicated NMR spectra of human urine samples, we repeated tests using synthetic samples containing either 10% D_2_O (used for internal NMR lock signal) or neat D_2_O (>99.8%), both with a pre-determined concentration of creatinine and DSS as the IS (Fig. 5 A–C and M–O). The synthetic samples not only provided simplified spectra for more accurate baseline integration/peak height comparisons (Fig. 5 A–F, M–R), but also permitted quantitation compared with known concentrations. The 100% D_2_O samples (at very low gain) were devised to provide a control baseline quantitation in the absence of all saturation pulses. However, we found that even with high concentrations of D_2_O, the water hydrogen signals were too intense and the receiver gain had to be minimized (Fig. 5 D–F, P–R). Disappointingly, in most cases some very weak saturation (*e.g.*, 5–20 Hz for 990 ms) was required. While this reduced the effectiveness of the control, the results still clearly showed that saturation perturbed resonances in a frequency-dependent manner as resonances approached the solvent (water) carrier position.

We also tested two distinct human volunteer urine samples, both with a known concentration of added DSS and one sample with a relatively high urea concentration (Fig. 5 G–I and S–U), and the other volunteer sample with a lower urea concentration (Fig. 5 J–L and V–X). We were then able to test results with “low” saturation power (∼20 Hz induced field strength or γB_1_), “normal” power (∼80 Hz), and “high” power (160–200 Hz). The reader should note that while we have classified the 200 Hz saturation power as “high,” this is of course several orders of magnitude lower than modern spectrometer hardware limitations (*e.g.*, 90° excitation pulses are delivered typically in the 20–40 kHz induced field strength range) and is high only in the common water saturation sense. Modern instruments should be able to deliver 200 Hz of induced field power continuously with extreme precision for very long periods of time (*i.e.*, seconds). We found that a similar metabolite peak suppression phenomenon occurred near the position of the solvent peak regardless of the concentration of urea in the sample, suggesting that metabolite peak suppression was only dependent on the saturation power applied rather than metabolites such as urea within each sample (Fig. 5).

## Discussion

It is our conclusion that quantitative metabolomics data can be accurately and precisely characterized at multiple acquisition venues, but that consistent data are far more sensitive to parameter settings associated with solvent suppression than previously reported. Perturbation of NMR signals resonating close to the solvent peak position, or originating from or near exchangeable atoms (*e.g.*, -OH or -NH groups or their near neighbors) have been known for quite some time [43], but the effects on the quantitation of resonances 1 to 2 PPM away due to solvent (water) suppression saturation has not been detailed for metabolomics data. Moreover, our findings suggest that 3 mm NMR probes are more sensitive to saturation power effects than 5 mm probes.

Quantitative NMR urine metabolomics is becoming a widely employed approach in systems biology science for biomarker discovery [10], [44], [45]. Like genomics and proteomics, due diligence in this process is also warranted for metabolomics, and should include cross-center analytical validation studies [15], [16]. The data presented here illustrate the importance of this principle because they show that small changes in saturation power levels, from, for example, 80 Hz- to 160 Hz-induced field strength, had a statistically significant and frequency-dependent effect on measured signal intensities that influenced metabolite quantification. Specifically, as metabolite resonances approached the water peak, the signal intensity of each respective resonance was reduced, while atoms from the same molecule resonating further away from the solvent position were less perturbed. Our findings strongly indicate that frequency-dependent calibration may be required when comparing data collected on different instruments utilizing different accepted solvent saturation powers. A recent study has shown that major changes to pulse sequences will result in changes to peak amplitudes [26]. Therefore, different facilities that use different NMR probes, must be extremely diligent to insure that actual delivered power levels, and not just the corresponding parameter values, are identical. We recommend that this be done prior to data collection with an oscilloscope and/or via the spectrometer itself, since NMR spectrometers are among the most accurate tools for measuring power delivery and response. A standard sample such as the demonstrated creatinine with DSS as an IS should suffice for calibrations.

While the indirect effects of solvent saturation on exchangeable functional groups and the surrounding “bleaching” effect on non-exchangeable resonances near the solvent have been well documented in biomolecular NMR spectroscopy [30], [43], [46], [47], the extent of perturbation, and the sensitivity of metabolomics quantitation to even small changes in saturation power, have not been reported for metabolomics studies. The results we present here show that the Chenomx and the literature-recommended value of saturation at ∼80 Hz γB_1_ induced field strength [48] is often sufficient for adequate solvent (water) suppression using the 1D-NOESY, and presents a narrow band of bleaching around the spectrometer carrier frequency. If the saturation power is insufficient for solvent suppression without causing bleaching, then a detailed power delivery along with quantitation effect(s) would be necessary to correct or even normalize data for future comparisons. This is highlighted by our observation that even small changes in saturation power had significant negative effects on neighboring resonance intensities. Our data also show (Fig. S4) significant accuracy and precision errors for measured resonances 1 PPM away, and even as far away as 2 PPM from the water frequency, and that solvent suppression power cannot simply be continually lowered in order to obtain better metabolite information. This is because even with “low” saturation power settings (∼20 Hz), perturbations were still evident. Surprisingly, peaks obtained with our lowest saturation power levels did not show proper NMR peak intensity ratios. This indicates that saturation powers lower than 80 Hz should be used; however, we experienced many problems (*e.g.* analog to digital conversion errors, receiver overloads, *etc.*) acquiring spectra with sufficiently suppressed solvent signals. While the spectrometer signal gain could be lowered, issues with the dynamic range of metabolite concentrations quickly came into play. This concept is demonstrated by the quantification of choline and acetate, both of which are low abundant metabolites in urine. In general, quantification of choline is more difficult and less reliable than acetate, because it has three low intensity peaks, two of which lie in the water suppression region (3.507 and 4.058 PPM) while acetate is a single, high intensity peak at 1.90 PPM. As such, compounds with overlapped or multiple, low intensity NMR peaks may be more difficult to assess. However, the majority of the 59 detected urinary metabolites (98%) showed significant correlation between the two sites of NMR data acquisition (Fig. 2 and Fig. S2).

The impact of saturation power settings on spectral acquisition was more pronounced using a 3 mm probe than it was using a 5 mm probe. These experiments were repeated on both 3 mm and 5 mm probes at the same site, using the same operator, samples, instrument, and day on which spectra were collected in order to remove as many variables as possible. Of special interest was that the ratio of metabolite peak height to DSS peak height decreased as saturation power was lowered. We had expected full relative peak intensities to be recovered by the “medium” saturation power. This was not the case, as significant magnitude changes even on “low” saturation settings were observed. This was most pronounced on 3 mm probes, and in order to corroborate our findings, a verification sample was sent to the University of Toronto (3 mm Agilent “One-Probe”) which generated data in agreement with our results (Fig. S5).

We next employed synthetic samples of creatinine (2 mM) with DSS in either 100% D_2_O or 10% D_2_O to confirm our findings. An advantage of these samples compared with actual human urine samples, was that they did not contain urea, an abundant urine metabolite [49]. Urea is excreted in highly variable amounts which is subjected to many factors such as hydration, diet, exercise, and injury or disease [50], [51]. In this study, we took special care to control for these factors with the exception of hydration, which was encouraged but not controlled. Overall, subjects were well hydrated as evidenced by urine specific gravity values between 1.005 and 1.015 (Table S1) with the exception of one subject whose urine had a specific gravity of 1.100 along with a high urea concentration [52]. This is relevant because urea can act as a large pool of non-suppressed hydrogen atoms, resonating downfield of water (∼5.78 PPM at 25°C not correcting for deuterium concentration effects or pH). To date, urine metabolomics studies have typically utilized single frequency saturation and therefore have not actively suppressed both the urea and the water signals [53]. Hydrogen atoms from urea readily exchange with the saturated (suppressed) hydrogen atoms from water. In essence, urea can act as a massive heat-sink for the relaxation of water resonances. The exchange rates of hydrogen into and out of water and urea result in very broad water and urea NMR signals. By not including urea in the synthetic test sample, we removed this vastly complex system. However, when we tested two human urine samples, one with a relatively high urea concentration and the other with one of the lowest urea levels, from our volunteers, we found that the presence of urea did not appreciably change the profile of perturbation caused by the saturation power. Since these are just two samples with widely divergent urea concentrations, additional studies of the influence of urea concentration on saturation are warranted, particularly since urine has far higher urea levels compared with other biofluids such as blood, serum or plasma.

In summary, our data lead us to suspect that the observed signal intensity perturbation may be widespread and previously unrecognized. For example, we found that the Human Metabolomics Database (HMDB - http:www.hmdb.ca) [41] shows the same peak integration error for creatinine (HMDB00562) as the one described here. As has been reported [24], [26], [34], [54] and clearly detailed by Chenomx, Inc. (Edmonton, Alberta, Canada), the entire Chenomx metabolite database and all its associated resonances have been collected using an exceedingly precisely arranged NMR pulse sequence. This sequence requires specific delays in order to maintain peak amplitude ratios, and adherence to specified delays is required for the proper function of the software's metabolite quantitation tool. The Chenomx database was established to empirically determine the ratios of peak intensities considering non-equilibrium spectra. Different functional groups and different molecular mobility are therefore compensated by directly measuring each metabolite prior to inclusion in the database. Undoubtedly, the HMDB uses a similar empirical approach for establishing expected peak ratios. However, these ratios appear to be dependent on timings that are relatively well understood and reported in the literature, but also now appear to be dependent on the saturation settings.

To the best of our knowledge, this is the first report of the dependency of peak ratios on saturation settings. This finding is significant because it highlights the importance and impact of saturation parameter settings on quantitative NMR metabolomics. We have not yet determined if this also applies to other biofluids, but we think this is highly likely due to the necessity for solvent saturation. We also expect that most single-center studies to date have been conducted with consistent NMR parameters, so intra-study comparisons should still be valid despite obvious potential problems with absolute reported quantitation. Specifically, the precision should be intact, but accuracy may be at risk, which does have implications for larger, cross-center studies that are likely to be conducted as part of biomarker discovery efforts. As such, there is a need for heightened awareness and attention to ensuring that saturation settings are consistent and uniform so that reliable, reproducible quantified metabolite data are generated.

## Conclusion

We have determined that 3 mm and 5 mm NMR probes commonly employed in metabolomics studies are both highly susceptible to saturation power levels when utilizing the common 1D-^1^H-NOESY NMR pulse sequence. While other common parameters such as excitation pulse width, spectrometer carrier position, and NMR lock gain settings are capable of small errors without dramatic changes in metabolite quantitation, it is our recommendation that in order to insure reliable in and cross-project comparisons of quantified metabolomics data, spectrometer parameters should be kept absolutely consistent at each site and importantly, each instrument should be calibrated and the appropriate equivalent settings determined in advance of conducting assays in cross-center studies. It is not sufficient to have the same setting values, and it is necessary to determine the actual delivered powers as each spectrometer is slightly different. Saturation power levels should be kept at 10–100 Hz induced field strength, noting that the use of slightly higher powers (*e.g.*, 160–320 Hz) will cause substantial and increasing perturbation in metabolite peaks as they approach the solvent (water) carrier position. However, this power level may not be sufficient for water suppression for high Q-factor NMR probes. In these cases, the receiver gain may have to be reduced, or a different solvent suppression system may have to be employed for the entire project. For any studies done prior to our findings described here, a calibration profile should be established using standardization samples to determine the envelope of saturation effect using the probe and spectrometer for the individual studies.

## Supporting Information

Figure S1 **Different creatinine and urea concentrations were acquired using a 3 mm and 5 mm NMR probe.**
(DOCX)

Figure S2 **There was a high degree of correlation between normalized metabolite concentrations obtained from the University of Alberta's and the University of Michigan's 5 mm probes.** Linear regression plots (Pearson) with associated 95% prediction bands (dashed lines) of normalized urine metabolites from ^1^H-NMR spectra acquired using 5 mm probes at the University of Michigan (UM) and at the University of Alberta (UA).
(DOCX)

Figure S3 **Box and whisker plots of the 59 urine metabolites quantified from ^1^H-NMR spectra acquired from technical replicate samples using a 5 mm probe (University of Alberta; UA), and a 3 and 5 mm probe at the University of Michigan (UM).**
(DOCX)

Figure S4 **Representation of several NMR peak amplitudes relative to DSS (100%).** Peaks were selected based on their frequency separations from the saturation carrier (∼4.7 PPM).
(DOCX)

Figure S5 **Peak heights from several 3 mm probes that were first normalized to DSS (100%) and then divided by the average amplitude of 4 separate 5 mm probe measurements.**
(DOCX)

Table S1 **Chemstrip 10 MD and pH data of NMR-assayed urine samples.**
(DOCX)

Text S1 **Standard operating procedure for urine collection and processing.**
(DOCX)

## References

[1] Nicholson JK, Lindon JC (2008) Systems biology: Metabonomics. Nature 455: 1054–1056. 18948945 10.1038/4551054a

[2] Psychogios N, Hau DD, Peng J, Guo AC, Mandal R, et al (2011) The human serum metabolome. PloS one 6: e16957. 21359215 10.1371/journal.pone.0016957 PMC3040193

[3] Bouatra S, Aziat F, Mandal R, Guo AC, Wilson MR, et al (2013) The human urine metabolome. PloS one 8: e73076. 24023812 10.1371/journal.pone.0073076 PMC3762851

[4] Dunn WB, Broadhurst DI, Atherton HJ, Goodacre R, Griffin JL (2011) Systems level studies of mammalian metabolomes: the roles of mass spectrometry and nuclear magnetic resonance spectroscopy. Chem Soc Rev 40: 387–426. 20717559 10.1039/b906712b

[5] Foxall PJ, Parkinson JA, Sadler IH, Lindon JC, Nicholson JK (1993) Analysis of biological fluids using 600 MHz proton NMR spectroscopy: application of homonuclear two-dimensional J-resolved spectroscopy to urine and blood plasma for spectral simplification and assignment. J Pharm Biomed Anal 11: 21–31. 8466956 10.1016/0731-7085(93)80145-q

[6] Xia J, Bjorndahl TC, Tang P, Wishart DS (2008) MetaboMiner–semi-automated identification of metabolites from 2D NMR spectra of complex biofluids. BMC Bioinformatics 9: 507. 19040747 10.1186/1471-2105-9-507 PMC2612014

[7] Serkova NJ, Brown MS (2012) Quantitative analysis in magnetic resonance spectroscopy: from metabolic profiling to in vivo biomarkers. Bioanalysis 4: 321–341. 22303835 10.4155/bio.11.320

[8] Griffin JL (2003) Metabonomics: NMR spectroscopy and pattern recognition analysis of body fluids and tissues for characterisation of xenobiotic toxicity and disease diagnosis. Curr Opin Chem Biol 7: 648–654. 14580571 10.1016/j.cbpa.2003.08.008

[9] Heather LC, Wang X, West JA, Griffin JL (2013) A practical guide to metabolomic profiling as a discovery tool for human heart disease. J Mol Cell Cardiol 55: 2–11. 23231771 10.1016/j.yjmcc.2012.12.001

[10] Slupsky CM, Cheypesh A, Chao DV, Fu H, Rankin KN, et al (2009) Streptococcus pneumoniae and Staphylococcus aureus pneumonia induce distinct metabolic responses. J Proteome Res 8: 3029–3036. 19368345 10.1021/pr900103y

[11] Nagrath D, Caneba C, Karedath T, Bellance N (2011) Metabolomics for mitochondrial and cancer studies. Biochim Biophys Acta 1807: 650–663. 21420931 10.1016/j.bbabio.2011.03.006

[12] Slupsky CM, Steed H, Wells TH, Dabbs K, Schepansky A, et al (2010) Urine metabolite analysis offers potential early diagnosis of ovarian and breast cancers. Clin Cancer Res 16: 5835–5841. 20956617 10.1158/1078-0432.CCR-10-1434

[13] Stringer KA, Serkova NJ, Karnovsky A, Guire K, Paine R 3rd, et al (2011) Metabolic consequences of sepsis-induced acute lung injury revealed by plasma (1)H-nuclear magnetic resonance quantitative metabolomics and computational analysis. Am J Physiol Lung Cell Mol Physiol 300: L4–L11. 20889676 10.1152/ajplung.00231.2010 PMC3023293

[14] Lacy P (2011) Metabolomics of sepsis-induced acute lung injury: a new approach for biomarkers. Am J Physiol Lung Cell Mol Physiol 300: L1–3. 21056960 10.1152/ajplung.00375.2010

[15] Dumas ME, Maibaum EC, Teague C, Ueshima H, Zhou B, et al (2006) Assessment of analytical reproducibility of 1H NMR spectroscopy based metabonomics for large-scale epidemiological research: the INTERMAP Study. Anal Chem 78: 2199–2208. 16579598 10.1021/ac0517085 PMC6561113

[16] Barton RH, Nicholson JK, Elliott P, Holmes E (2008) High-throughput 1H NMR-based metabolic analysis of human serum and urine for large-scale epidemiological studies: validation study. Int J Epidemiol 37 Suppl 1: i31–40. 18381391 10.1093/ije/dym284

[17] Keun HC, Ebbels TM, Antti H, Bollard ME, Beckonert O, et al (2002) Analytical reproducibility in (1)H NMR-based metabonomic urinalysis. Chem Res Toxicol 15: 1380–1386. 12437328 10.1021/tx0255774

[18] Ioannidis JP, Khoury MJ (2011) Improving validation practices in “omics” research. Science 334: 1230–1232. 22144616 10.1126/science.1211811 PMC5624327

[19] Koulman A, Lane GA, Harrison SJ, Volmer DA (2009) From differentiating metabolites to biomarkers. Anal Bioanal Chem 394: 663–670. 19277615 10.1007/s00216-009-2690-3 PMC2865640

[20] Poste G (2011) Bring on the biomarkers. Nature 469: 156–157. 21228852 10.1038/469156a

[21] Anonymous (2000) World Medical Association declaration of Helsinki - Ethical principles for medical research involving human subjects. Jama-Journal of the American Medical Association 284: 3043–3045. 11122593

[22] Park Y, Kim SB, Wang B, Blanco RA, Le NA, et al (2009) Individual variation in macronutrient regulation measured by proton magnetic resonance spectroscopy of human plasma. Am J Physiol Regul Integr Comp Physiol 297: R202–209. 19458279 10.1152/ajpregu.90757.2008 PMC2711699

[23] McKay RT (2009) Recent advances in solvent suppression for solution NMR: A practical reference. In: Webb GA, editor. Annual Reports on NMR Spectroscopy. London: Elsevier. pp. 33–74.

[24] McKay RT (2011) How the 1D-NOESY Suppresses Solvent Signal in Metabonomics NMR Spectroscopy: An Examination of the Pulse Sequence Components and Evolution. Concept Magn Reson A 38A: 197–220.

[25] McKay RT, Mercier P, Sykes BD (2009) A comparative analysis of solvent suppression techniques and improvements for high resolution 1H NMR metabonomics studies. 50th Experimental Nuclear Magnetic Resonance Conference. Asilomar Conference Grounds, Pacific Grove, CA USA. Available: http://www.enc-conference.org. Accessed 2013 Aug 8.

[26] Sololenko S, McKay R, Blondeel EJM, Lewis MJ (2013) Understanding the variability of compound quantification from targeted profilling metabolomics of 1D-1H-NMR spectra in synthetic mixtures and urine with additional insights on choice of pulse sequences and robotic sampling. Metabolomics Epub ahead of print Feb 14, 2013. doi:10.1007/s11306-013-0503-3

[27] Wu PS, Otting G (2005) Rapid pulse length determination in high-resolution NMR. J Magn Reson 176: 115–119. 15972263 10.1016/j.jmr.2005.05.018

[28] Keifer PA (1999) 90 degrees pulse width calibrations: How to read a pulse width array. Concept Magnetic Res 11: 165–180.

[29] Reynolds WF, Enriquez RG (2002) Choosing the best pulse sequences, acquisition parameters, postacquisition processing strategies, and probes for natural product structure elucidation by NMR spectroscopy. J Nat Prod 65: 221–244. 11858762 10.1021/np010444o

[30] Mo H, Harwood JS, Raftery D (2010) Receiver gain function: the actual NMR receiver gain. Magn Reson Chem 48: 235–238. 20063326 10.1002/mrc.2563 PMC3071559

[31] Shishmarev D, Otting G (2011) Radiation damping on cryoprobes. J Magn Reson 213: 76–81. 21955524 10.1016/j.jmr.2011.08.040

[32] Gregory RM, Bain AD (2009) The Effects of Finite Rectangular Pulses in NMR: Phase and Intensity Distortions for a Spin-1/2. Concept Magn Reson A 34A: 305–314.

[33] Jahnke W, Kessler H (1994) Enhanced sensitivity of rapidly exchanging amide protons by improved phase cycling and the constructive use of radiation damping. J Biomol NMR 4: 735–740. 7919957 10.1007/BF00404281

[34] Mercier P, Lewis MJ, Chang D, Baker D, Wishart DS (2011) Towards automatic metabolomic profiling of high-resolution one-dimensional proton NMR spectra. J Biomolec NMR 49: 307–323. 10.1007/s10858-011-9480-x 21360156

[35] van den Berg RA, Hoefsloot HC, Westerhuis JA, Smilde AK, van der Werf MJ (2006) Centering, scaling, and transformations: improving the biological information content of metabolomics data. BMC genomics 7: 142. 16762068 10.1186/1471-2164-7-142 PMC1534033

[36] Kohl SM, Klein MS, Hochrein J, Oefner PJ, Spang R, et al (2012) State-of-the art data normalization methods improve NMR-based metabolomic analysis. Metabolomics 8: 146–160. 22593726 10.1007/s11306-011-0350-z PMC3337420

[37] Ludbrook J (2008) The presentation of statistics in Clinical and Experimental Pharmacology and Physiology. Clin Exp Pharmacol Physiol 35: 1271–1274 author reply 1274. 18954331 10.1111/j.1440-1681.2008.05003.x

[38] Curran-Everett D, Benos DJ (2004) Guidelines for reporting statistics in journals published by the American Physiological Society. Am J Physiol Endocrinol Metab 287: E189–191. 15271643 10.1152/ajpendo.00213.2004

[39] White BC, Jamison KM, Grieb C, Lally D, Luckett C, et al (2010) Specific gravity and creatinine as corrections for variation in urine concentration in humans, gorillas, and woolly monkeys. Am J Primatol 72: 1082–1091. 20648576 10.1002/ajp.20867

[40] Warrack BM, Hnatyshyn S, Ott KH, Reily MD, Sanders M, et al (2009) Normalization strategies for metabonomic analysis of urine samples. J Chromatogr B Analyt Technol Biomed Life Sci 877: 547–552. 10.1016/j.jchromb.2009.01.007 19185549

[41] Garde AH, Hansen AM, Kristiansen J, Knudsen LE (2004) Comparison of uncertainties related to standardization of urine samples with volume and creatinine concentration. Ann Occup Hyg 48: 171–179. 14990438 10.1093/annhyg/meh019

[42] Wishart DS, Jewison T, Guo AC, Wilson M, Knox C, et al (2013) HMDB 3.0–The Human Metabolome Database in 2013. Nucleic Acids Res 41: D801–807. 23161693 10.1093/nar/gks1065 PMC3531200

[43] Grzesiek S, Bax A (1993) The Importance of Not Saturating H2o in Protein Nmr - Application to Sensitivity Enhancement and Noe Measurements. J Am Chem Soc 115: 12593–12594.

[44] Nevedomskaya E, Pacchiarotta T, Artemov A, Meissner A, van Nieuwkoop C, et al (2012) (1)H NMR-based metabolic profiling of urinary tract infection: combining multiple statistical models and clinical data. Metabolomics 8: 1227–1235. 23136561 10.1007/s11306-012-0411-y PMC3483096

[45] McClay JL, Adkins DE, Isern NG, O'Connell TM, Wooten JB, et al (2010) (1)H nuclear magnetic resonance metabolomics analysis identifies novel urinary biomarkers for lung function. J Prot Res 9: 3083–3090. 10.1021/pr1000048 20408573

[46] Hoult DI (1976) Solvent Peak Saturation with Single-Phase and Quadrature Fourier Transformation. J Magn Reson 21: 337–347.

[47] Gueron M, Plateau P, Decorps M (1991) Solvent Signal Suppression in NMR. Prog Nucl Mag Res Sp 23: 135–209.

[48] Mo H, Raftery D (2008) Pre-SAT180, a simple and effective method for residual water suppression. J Magn Reson 190: 1–6. 17945521 10.1016/j.jmr.2007.09.016 PMC2662483

[49] Orita Y, Urakabe S, Shirai D, Furukawa T, Abe H (1966) Effect of urinary urea/nonurea: on urinary concentrating ability and renal water economy in human subjects. Jpn Circ J 30: 225–235. 5952243 10.1253/jcj.30.225

[50] Spector DA, Yang Q, Wade JB (2007) High urea and creatinine concentrations and urea transporter B in mammalian urinary tract tissues. Am J Physiol Renal Physiol 292: F467–474. 16849692 10.1152/ajprenal.00181.2006

[51] Yang B, Bankir L (2005) Urea and urine concentrating ability: new insights from studies in mice. Am J Physiol Renal Physiol 288: F881–896. 15821253 10.1152/ajprenal.00367.2004

[52] Pradella M, Dorizzi RM, Rigolin F (1988) Relative density of urine: methods and clinical significance. Crit Rev Clin Lab Sci 26: 195–242. 3077030 10.3109/10408368809105890

[53] Kupce E, Freeman R (1993) Techniques for Multisite Excitation. J Magn Reson Series A 105: 234–238.

[54] Weljie AM, Newton J, Mercier P, Carlson E, Slupsky CM (2006) Targeted profiling: quantitative analysis of 1H NMR metabolomics data. Anal Chem 78: 4430–4442. 16808451 10.1021/ac060209g

