# Correction: Signal Intensities Derived from Different NMR Probes and Parameters Contribute to Variations in Quantification of Metabolites

**DOI:** 10.1371/journal.pone.0102929

**Published:** 2014-07-10

**Authors:** The PLOS ONE Staff

The affiliation for the second author is not indicated. Ryan T. McKay is affiliated with: Department of Chemistry, University of Alberta, Edmonton, Alberta, Canada.

## References

[1] Lacy P, McKay RT, Finkel M, Karnovsky A, Woehler S, et al (2014) Signal Intensities Derived from Different NMR Probes and Parameters Contribute to Variations in Quantification of Metabolites. PLoS ONE 9(1): e85732 doi:10.1371/journal.pone.0085732 24465670 10.1371/journal.pone.0085732 PMC3897511

