# Supplementary material for: Signal Intensities Derived from Different NMR Probes and Parameters Contribute to Variations in Quantification of Metabolites
Source: PLoS One. 2014 Jan 21;9(1):e85732. doi: 10.1371/journal.pone.0085732 (PMC3897511; doi:10.1371/journal.pone.0085732)
Supplement: Figure S1 — Different creatinine and urea concentrations were acquired using a 3 mm and 5 mm NMR probe. (DOCX) [file pone.0085732.s001.docx]

** Figure S1**: Different creatinine and urea concentrations were acquired using a 3mm and 5mm NMR probe. The mean (+S.D.) concentrations of urine creatinine and urea quantified from ^1^H-NMR spectra of technical replicate samples from healthy volunteers (n=19) acquired at the University of Alberta (UA) and the University of Michigan (UM) using a 5mm and 3mm probe, respectively. The UA creatinine and urea concentrations were different from the UM creatinine and urea concentrations (*P* = 0.017 and *P* = 0.010, respectively, by Mann-Whitney test).
