# Supplementary material for: Signal Intensities Derived from Different NMR Probes and Parameters Contribute to Variations in Quantification of Metabolites
Source: PLoS One. 2014 Jan 21;9(1):e85732. doi: 10.1371/journal.pone.0085732 (PMC3897511; doi:10.1371/journal.pone.0085732)
Supplement: Figure S2 — There was a high degree of correlation between normalized metabolite concentrations obtained from the University of Alberta's and the University of Michigan's 5 mm probes. Linear regression plots (Pearson) with associated 95% prediction bands (dashed lines) of normalized urine metabolites from 1H-NMR spectra acquired using 5 mm probes at the University of Michigan (UM) and at the University of Alberta (UA). (DOCX) [file pone.0085732.s002.docx]

**Figure S2**: There was a high degree of correlation between normalized metabolite concentrations obtained from the University of Alberta’s and the University of Michigan’s 5mm probes. Linear regression plots (Pearson) with associated 95% prediction bands (dashed lines) of healthy volunteer (n = 19) normalized urine metabolites from ^1^H-NMR spectra acquired using 5 mm probes at the University of Michigan (UM) and at the University of Alberta (UA). In all cases, the p value was < 0.05.
