# Supplementary material for: Signal Intensities Derived from Different NMR Probes and Parameters Contribute to Variations in Quantification of Metabolites
Source: PLoS One. 2014 Jan 21;9(1):e85732. doi: 10.1371/journal.pone.0085732 (PMC3897511; doi:10.1371/journal.pone.0085732)
Supplement: Figure S3 — Box and whisker plots of the 59 urine metabolites quantified from 1H-NMR spectra acquired from technical replicate samples using a 5 mm probe (University of Alberta; UA), and a 3 and 5 mm probe at the University of Michigan (UM). (DOCX) [file pone.0085732.s003.docx]

**Figure S3:** Box and whisker plots of the 59 urine metabolites quantified from ^1^H-NMR spectra acquired from technical replicate samples using a 5mm probe (University of Alberta; UA), and a 3 and 5mm probe at the University of Michigan (UM). The lower and upper ends of each box represent the 25^th^ and 75^th^ percentiles, respectively, and the crossbar is the median concentration. The whiskers represent the minimum and maximum values.
