# Supplementary material for: Signal Intensities Derived from Different NMR Probes and Parameters Contribute to Variations in Quantification of Metabolites
Source: PLoS One. 2014 Jan 21;9(1):e85732. doi: 10.1371/journal.pone.0085732 (PMC3897511; doi:10.1371/journal.pone.0085732)
Supplement: Figure S4 — Representation of several NMR peak amplitudes relative to DSS (100%). Peaks were selected based on their frequency separations from the saturation carrier (∼4.7 PPM). (DOCX) [file pone.0085732.s004.docx]

**Figure S4**: Representation of several NMR peak amplitudes relative to DSS (100%). Peaks were selected based on their frequency separations from the saturation carrier (~4.7 PPM). Identical samples were measured on 5 mm (blue) and 3 mm (red) probes utilizing low (darkest colors), normal (middle colors), and high (lightest color) saturation powers. Two different peaks were selected for the downfield region to insure that an individual molecule’s relaxation rate was not playing a role in the result. Two gain settings (middle colors) were utilized to exclude any receiver dependencies. This figure shows significant amplitude perturbations based on saturation power at several PPM from the carrier. Saturation powers of 20 Hz, 80 Hz and 200 Hz (γB_1_) represent low, medium, and high, respectively. In addition to the high urea urine sample data shown above, a low urea urine sample, and two comparative synthetic samples containing creatinine and DSS (internal standard) dissolved in either 99.9% D2O, or 10% D2O were also analyzed.  These additional samples showed the same trends regarding the frequency dependent amplitude perturbation.
