# Supplementary material for: Signal Intensities Derived from Different NMR Probes and Parameters Contribute to Variations in Quantification of Metabolites
Source: PLoS One. 2014 Jan 21;9(1):e85732. doi: 10.1371/journal.pone.0085732 (PMC3897511; doi:10.1371/journal.pone.0085732)
Supplement: Figure S5 — Peak heights from several 3 mm probes that were first normalized to DSS (100%) and then divided by the average amplitude of 4 separate 5 mm probe measurements. (DOCX) [file pone.0085732.s005.docx]

**Figure S5:** Peak heights from several 3 mm probes that were first normalized to DSS (100%) and then divided by the average amplitude of 4 separate 5 mm probe measurements. The effect of high power saturation (dark green) can be seen compared to lower power (light green). The red and yellow bars show the result of using a pulse sequence that does not utilize saturation for water suppression. No frequency dependency was found when saturation was not used. The initial 3 mm probe data (blue) show the largest perturbation as the observed peaks approach the solvent position. Mich = University of Michigan; UofT = University of Toronto.
