# Supplementary material for: Signal Intensities Derived from Different NMR Probes and Parameters Contribute to Variations in Quantification of Metabolites
Source: PLoS One. 2014 Jan 21;9(1):e85732. doi: 10.1371/journal.pone.0085732 (PMC3897511; doi:10.1371/journal.pone.0085732)
Supplement: Table S1 — Chemstrip 10 MD and pH data of NMR-assayed urine samples. (DOCX) [file pone.0085732.s006.docx]

**Supplementary Table 1:** Chemstrip 10 MD and pH data of NMR-assayed urine samples

| **SAMPLE:** | 1 | 2 | 3 | 4 | 5 | 6 | 7 | 8 | 9 | 10 | 11 | 12 | 13 | 14 | 15 | 16 | 17 | 18 | 19 |
| --- | --- | --- | --- | --- | --- | --- | --- | --- | --- | --- | --- | --- | --- | --- | --- | --- | --- | --- | --- |
| Specific Gravity | 1.005 | 1.010 | 1.010 | 1.005 | 1.005 | 1.005 | 1.005 | 1.015 | 1.100 | 1.015 | 1.005 | 1.005 | 1.005 | 1.010 | 1.010 | 1.005 | 1.010 | 1.005 | 1.005 |
| Leukocytes | N | T | N | N | N | N | N | N | N | N | N | N | N | N | N | N | N | N | N |
| Nitrite | N | N | N | N | N | N | N | N | N | N | N | N | N | N | N | N | N | N | N |
| Proteins | T | T | T | T | T | T | T | T | T | T | T | T | T | T | T | low T | T | T | low T |
| Glucose | NL | NL | NL | NL | NL | NL | NL | NL | NL | NL | NL | NL | NL | NL | NL | NL | NL | NL | NL |
| Ketones | N | N | N | N | N | N | N | N | N | N | N | N | N | N | N | N | +  Small | N | N |
| Urobilinogen | NL | NL | NL | NL | NL | NL | NL | NL | NL | NL | NL | NL | NL | NL | NL | NL | NL | NL | NL |
| Bilirubin | N | N | N | N | N | N | N | N | N | N | N | N | N | N | N | N | N | N | N |
| Blood | N | N | N | N | N | N | 50 ERy/ul | N | N | N | N | N | N | N | N | N | N | N | N |
| Final pH* | 6.78 | 7.04 | 7.25 | 7.11 | 7.22 | 6.88 | 7.04 | 6.86 | 7.01 | 6.96 | 6.97 | 7.13 | 7.02 | 7.01 | 7.25 | 6.89 | 6.99 | 7.24 | 7.22 |

N = negative; T = trace; NL = normal; + = positive; ERy = erthyrocytes; *at the time of storage
