# Supplementary material for: Signal Intensities Derived from Different NMR Probes and Parameters Contribute to Variations in Quantification of Metabolites
Source: PLoS One. 2014 Jan 21;9(1):e85732. doi: 10.1371/journal.pone.0085732 (PMC3897511; doi:10.1371/journal.pone.0085732)
Supplement: Text S1 — Standard operating procedure for urine collection and processing. (DOCX) [file pone.0085732.s007.docx]

**TEXT S1: Standard operating procedure employed for the study’s urine collection**

In advance and in preparation for sample collection, Becton Dickinson (BD) Vacutainer® urine collection cups (100 ml; cat no. **364975**) were coated with sodium azide (NaN_3_) by adding 100 μl of 10% NaN_3_ using aseptic technique in a biosafety cabinet and were allowed to dry at room temperature. Prepared cups were capped and stored in a sealed zip-lock plastic bag at 4°C for up to 6 months. In advance of urine collection, BD Vacutainer urinalysis tubes (8 ml; yellow top; no additive; cat no. 364980) were prepared by injecting 160 µl of 10% NaN_3_ into each need tube using a 1 ml syringe with a 23G needle (to avoid disruption of the tube’s vacuum).

On the day of collection, subjects were instructed not to exercise and to be well hydrated with a full bladder. Urine sample collection was planned for between 0830-0930 and patients were fasting (at least 12 h). Urine was collected as a midstream, clean-catch sample and at least 50 ml urine was collected into the NaN_3_ coated collection cup. The cup was immediately capped, wrapped in foil, and placed on ice or in the refrigerator (2-4ºC).

Within 3 h of collection, urine (8 ml) was transferred into a prepared BD Vacutainer® urinalysis tube using the integrated transfer device in the lid of the specimen cup. The tube was inverted eight times and then centrifuged (4°C, 2000 x *g*, 10 min) to precipitate sediment. Following centrifugation, the supernatant was transferred to a sterile conical tube (15 ml) taking care not to disturb the bottom of the tube whether there was a visible pellet or not. The remaining pellet and ~500 μl of sample above it were discarded. The sample was placed on ice and the pH and volume were measured and a volume of Chenomx standard solution (IS-1 Chenomx internal standard- DSS with added imidazole) equivalent to 10% of the final sample volume was added. Following the addition of IS-1, the tube was inverted several times to thoroughly mix. The sample was placed on ice and the pH was measured again. To achieve a pH of 7.0+0.25, NaOH (1 M), typically < 5-10 µl, was added. Once the needed pH was reached, keeping the sample on ice, 1 ml was transferred to a microcentrifuge tube (1.5 ml). This volume was dipsticked using a Chemstrip 10 MD (Roche Diagnostics Corporation, Indianapolis, IN; cat no. 03260763160) after which this aliquot was discarded. With tubes and the remaining sample on ice, technical replicates were generated by allocating 1ml of urine into labeled sterile microcentrifuge tubes (1.5 ml). Aliquots were frozen (-80°C) until the time of assay. The resulting data from the Chemstrip dipstick are shown in Table S1.
